# The Integration of Science and Policy in Regulatory Decision-Making: Observations on Scientific Expert Panels Deliberating GM Crops in Centers of Diversity

**DOI:** 10.3389/fpls.2018.01157

**Published:** 2018-08-08

**Authors:** Karen E. Hokanson, Norman Ellstrand, Alan Raybould

**Affiliations:** ^1^Department of Horticultural Science, University of Minnesota, St. Paul, MN, United States; ^2^Department of Botany and Plant Sciences, University of California, Riverside, Riverside, CA, United States; ^3^Regulatory Science, Syngenta Crop Protection AG, Basel, Switzerland

**Keywords:** GM crop regulation, risk assessment, problem formulation, risk characterization, expert panels, gene flow, centers of diversity

## Abstract

Panels of experts with specialized knowledge and experience are often convened to identify and analyze information relevant for risk assessments of GM crops. A perspective on the use of such scientific expert panels is shared here based on panels convened to inform the regulatory strategy for three separate projects developing GM crops for cultivation in Africa: a nutritionally enhanced sorghum, an insect resistant cowpea, and a virus resistant cassava. The panels were convened specifically to consider the risks associated with gene flow from a genetically modified (GM) crop to naturally occurring ‘wild’ relatives of that crop. In these cases, the experts used problem formulation to identify effects that regulatory authorities may consider to be harmful (“harms”) and formulate plausible scenarios that might lead to them, and the availability of information that could determine the likelihood of the steps in the pathway. These panels and the use of problem formulation worked well to gather the existing information and consider the likelihood of harm from gene flow in centers of diversity. However, one important observation from all of these cases is that it is outside the remit of such scientific expert panels to make decisions dependent on policy, such as which harms should be considered and what information should be considered essential in order for a regulatory authority to make a decision about the acceptable level of risk. These experiences of expert panels to inform GM crop risk assessment demonstrate the challenge of integrating science and policy for effective regulatory decision-making.

## Introduction

The cultivation of genetically modified (GM) crops can bring significant benefits to farmers, the environment and society (e.g., Klümper and Qaim, 2014; Brookes and Barfoot, 2017a,b). GM crops are also strictly regulated because of concerns that their use may have detrimental effects on human health and the environment (Jaffe, 2004). There is a long-standing concern, however, that ‘over-regulation’ of the products of biotechnology is preventing the realization of the benefits they offer, particularly in developing countries (e.g., Paarlberg, 2006). Central to this problem is the need to discern whether and how scientific and non-scientific evidence should be used in regulatory decision-making (Adenle et al., 2018). In particular, assessment of environmental risk is often hindered by the absence of clear policy objectives that are needed to guide the interpretation of scientific data (Evans et al., 2006).

In this perspective, we share observations from our experience with three separate scientific expert panels convened to inform risk assessments on the specific issue of gene flow from GM sorghum, cowpea, and cassava to wild plants in various parts of Sub-Saharan Africa. We found that such panel discussions and the use of problem formulation are first excellent forums for organizing existing knowledge in order to predict the likelihood of harmful effects, at least in these cases of transgene introgression into wild species, including in the crop’s centers of diversity; and second, useful for identifying scientific uncertainty associated with the predictions, and studies that could be conducted to reduce that uncertainty. In addition to these, the most notable observation on these expert panels is the need to discern scientific and non-scientific information, as was evident in these discussions. Outside the remit of such panels is decision-making responsibilities that include definitions of harm and judging the sufficiency of scientific knowledge and the extent to which uncertainties must be reduced for decision-making. Hence, the panels demonstrate the challenge of integrating scientific and non-scientific policy-related information in decision-making and the need for clear policy in order to avoid an unnecessary quest for more and more scientific information.

## The Problem: Assessing the Risks From Gene Flow to Wild Relatives

The potential for harmful effects following gene flow from GM crops to sexually compatible wild relatives was among the earliest environmental concerns associated with GM crops (e.g., Dale, 1992). Frameworks to assess the risks from gene flow to wild relatives are not defined as well as those to assess some other risks, such as the use of substantial equivalence for food safety (e.g., Novak and Haslberger, 2000; König et al., 2004), or the tiered approach that is used for non-target organism assessment (e.g., Garcia-Alonso et al., 2006; Romeis et al., 2008). Early gene flow studies were concerned more with the frequency and distance of gene flow (e.g., Timmons et al., 1995; Ellstrand et al., 1999; Ellstrand, 2003), although these studies rarely lead to a risk conclusion without a need to consider the consequences. It is more difficult and few attempts have been made to design frameworks and studies that assess the more complex questions about consequences of gene flow (although, see Snow et al., 2003; Raybould and Cooper, 2005; Sasu et al., 2010).

The crops which were the subject of the expert panel discussions included sorghum (*Sorghum bicolor*) and cowpea (*Vigna unguiculata* subsp. *unguiculata*), which are staple crops being developed for use in, or close to, their center of diversity in Africa. The center of diversity can be defined as the geographic area where there is a high level of *in situ* genetic diversity for a crop species. The third crop, cassava (*Manihot esculenta*), is a staple crop but its center of diversity is not in Africa; however, there is one known, introduced compatible wild (free-living) relative of cassava (*M. glaziovii*) found in Africa. These three crops are the subject of continuing research to use genetic engineering to introduce traits with the potential to dramatically improve value for farmers and consumers in Africa: sorghum with multiple genes for nutritional enhancement traits (increased vitamin A, iron, zinc, lysine, and threonine) in East and West Africa; cowpea producing Cry1Ab that confers resistance to the pod borer in West Africa; and cassava using RNA interference (RNAi) technology for cassava brown streak virus (CBSV) resistance in East Africa.

The first regulatory scrutiny of these and similar GM crops being developed by international non-profit, philanthropic or governmental development organizations, is likely to occur in countries where regulatory authorities have limited experience of and resources for evaluating the technology, including risk assessment. It is important, therefore, that risk assessments exploit existing knowledge and not default to requirements for new data when it is not necessary for effective decision-making, as is an unfortunate trend in cases where there are more resources. To this end, the first of these panels, comprising experts in risk assessment, gene flow, sorghum biology and sorghum as a crop in Africa was assembled in 2008 by the Africa Biofortified Sorghum project to discuss how to assess the risks from cultivating nutritionally enhanced sorghum in the center of sorghum diversity. Sorghum is a major crop in sub-Saharan Africa and its center of diversity is in Ethiopia and Sudan (Harlan, 1971); therefore, if GM sorghum is to be grown in Africa, the question of risks from gene flow in centers of origin and diversity has to be addressed. It is important to note that the panel was not asked to make a decision about the risks, but to share their experience and expertise within the framework of problem formulation and likelihood assessment.

The sorghum panel provided the template for the composition and method of working for the subsequent panels on cowpea and cassava in East Africa. Each panel selected by the projects consisted of approximately six scientific experts (not regulators), half being experts from Africa, who had expertise in the area of GM crop risk assessment, gene flow, or the biology and ecology of the crop and its relatives. Although other panel members differed among panels, the authors of this perspective participated on each of these panels, and the three discussions were facilitated by author K. Hokanson. The sorghum and cowpea panels met in St. Louis, MO, United States in 2008 and 2010, respectively; the cassava panel met in Mombasa, Kenya in 2015. Each panel more-or-less followed the process of problem formulation as outlined below. More details of these potential products and of the panel discussions are described in previous publications (sorghum: Hokanson et al., 2010; cowpea: Huesing et al., 2011; and cassava: Hokanson et al., 2016).

## Problem Formulation and Likelihood Assessment Using Existing Information

Problem formulation for assessing the risks from using GM crops may be regarded as a method for formulating and proposing tests of hypotheses that are relevant for making decisions concerning particular products (Raybould, 2006). At their most conservative, the hypotheses under test are similar for all crops: growing genetically modified crop X in region Y will not result in harmful effect Z. Less conservative hypotheses are that growing the crop poses no unacceptable risk. Corroboration of a hypothesis of no harm provides rigorous corroboration of a hypothesis of no unacceptable risk, whereas falsification of a hypothesis of no harm does not necessarily indicate unacceptable risk – the risk may be acceptable depending on, for example, the opportunities presented by cultivating the crop (Sanvido et al., 2012). Hypotheses of no harm or of no unacceptable risk are called “risk hypotheses.”

After problem formulation, the risks can be characterized by testing the risk hypotheses with existing information. ‘Testing’ a hypothesis does not necessarily require experimentation. If a hypothesis is corroborated or falsified using available existing information with sufficient certainty for decision-making, no further testing of that hypothesis ought to be necessary for the purposes of risk assessment; however, there may be interest in testing the hypothesis for other reasons. If a hypothesis requires further testing for decision-making, problem formulation devises testing through new studies or observations, or by gaining access to previously unavailable existing information. Because risk assessment is a decision-making tool, and not basic research, simple, rigorous tests of hypotheses under unrealistically conservative conditions are generally preferred. If a risk hypothesis is falsified under conservative testing, a further round of problem formulation may lead to a decision to conduct further testing under more realistic conditions, or to complete the risk assessment based on the conservative tests (Raybould, 2006).

The expert panels followed the principles of problem formulation outlined above as a means to gather and deliberate about existing information. First, the panels decided what effects of gene flow from the crop to a wild relative should might be regarded as environmental ‘harm’ by a regulator. (‘Harm’ in this sense is synonymous with ‘adverse effects’ as used in the methodology outlined for risk assessment under Annex III of the Cartagena Protocol on Biosafety.) Determination of which harms are to be considered in regulatory risk assessment is a matter of policy and would normally be derived from protection goals; that is, the overall objectives of the policy that the regulations are intended to deliver (e.g., Hommen et al., 2010; Garcia-Alonso and Raybould, 2014). As the panels were operating independently of any specific policy guidance, determination of harm was based on precedent from existing risk assessments and assumptions about change that might be regarded as detrimental to the environment, and as such represented an opinion of the experts rather than a regulatory determination. For these panel discussions, harms were defined necessarily to carry out the problem formulation; they were certainly not intended to influence the regulatory policy of any country.

**FIGURE 1:**
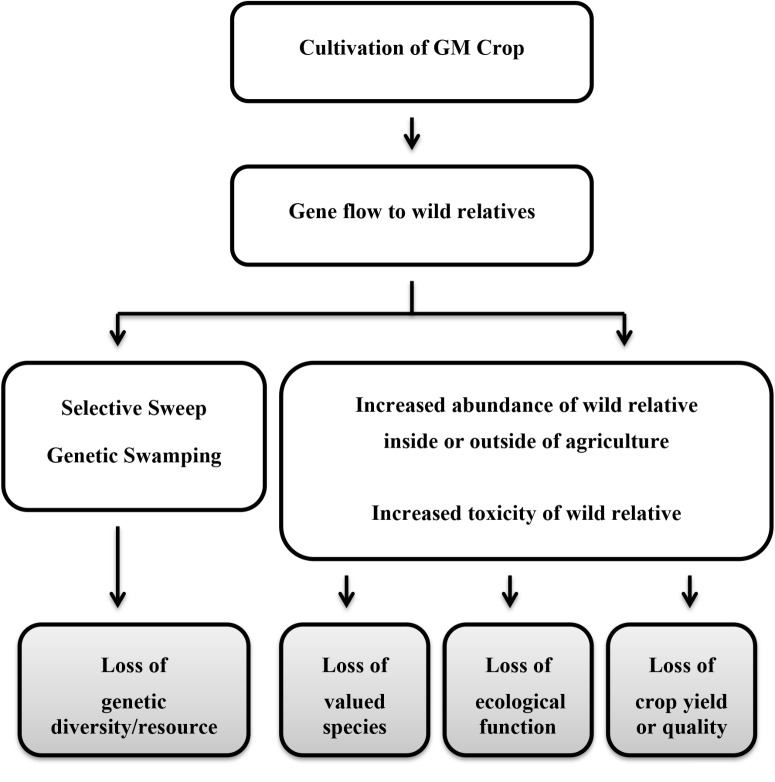
Simplified pathways to harm resulting from gene flow to a wild relative. Gray boxes indicate the harm (adverse effect).

The panels identified a similar list of harms to consider further for each of the crops, which can be summarized as arising from two basic mechanisms: (1) genetic changes resulting from selective sweeps or genetic swamping; and (2) demographic changes resulting from changes in species abundance or an increase in toxicity or decrease in nutritional quality of the wild relative (**Figure 1**). The harm due to the first mechanism would be a loss of valuable genetic diversity in the crop gene pool. From the second mechanism, harms included reduced abundance or diversity of valued species (native flora and fauna or domestic animals) or reduced crop yield or quality through loss of ecosystem services. Loss of valuable ecological functions underlying other ecosystem services was also postulated, but more precise harms were not specified. The panels recognized that the presence of transgenes in wild populations in the absence of any other genetic or demographic effects might be considered of concern on religious or cultural grounds; however, these harmful effects were not considered further as science has little or no contribution to characterizing risk in such circumstances other than to indicate whether or not gene flow is conceivable.

Defining the harms allowed the panels’ deliberations to concentrate on elaborating the steps that would need to occur for a harm to be realized; the series of steps leading to a particular harmful effect is called a “conceptual model” or “pathway to harm.” A highly simplified summary of the two principal pathways considered by the panels is depicted (**Figure 1**). Without this focus on defined harmful effects, it is likely that the panels would have attempted a comprehensive description of all possible effects following release of the particular GM crop, which is neither an efficient nor effective method of risk assessment.

Once the pathways to harm had been described, the likelihood of each step being realized was evaluated by the panels using existing knowledge. A likelihood assessment determines the degree of chance that harm, or a step leading to harm, occurs OGTR (2009). Ascribing a likelihood to a step, e.g., highly unlikely, unlikely, likely, is in effect a determination of the confidence in the corroboration or falsification of the hypothesis that the particular step in the pathway will not occur. In theory, once a single step in the pathway is deemed as highly unlikely with sufficient confidence, the risk via the pathway can be designated as negligible. However, just as harm and acceptable risk are matters for policy, so is the determination that a hypothesis has been corroborated with sufficient rigor. Hence, even though a particular step in a pathway is deemed unlikely, the discussion of subsequent steps usually continues so that risk could be determined as the cumulative probability of every step being realized in sequence. For each crop, the cumulative probability along each pathway suggests that harm was unlikely to occur via gene flow from the respective crop to its wild relative (Hokanson et al., 2010, 2016; Huesing et al., 2011).

## Problem Formulation and Likelihood Assessment in Centers of Diversity

In these panel discussions, the harmful effects as defined were the same whether or not the crop is grown in its center of diversity (as for sorghum and cowpea) or not (as for cassava), and the process of problem formulation and risk assessment is the same in all cases, although the pathways may be different and more or less probability may have been assigned to different steps. That is to say, different methodology was not necessary to conduct risk assessment for a GM crop in the center of diversity. When conducting risk assessment for GM crops in centers of diversity, the most important thing is to define the harmful effects at the outset. In centers of diversity the primary (although not the only) concern is likely to be the protection of a genetic resource. If this is the case, it is essential that a plausible mechanism by which that harm may arise from growing the specific GM crop is set out, as it was in the panel deliberations.

The harms and the pathways that can lead to their manifestation as defined by the expert panels are a useful start for any discussion of the risks of crop to wild relative gene flow. Particularly in centers of diversity, a loss of diversity in the gene pool of the crop resulting from gene flow has an increased probability than elsewhere simply because compatible wild relatives and a high level of valuable diversity are usually found in a crop’s center of diversity. The cassava panel used their knowledge about the center of diversity for cassava to determine that ‘loss of genetic diversity’ is unlikely if the GM cassava is grown in Africa because valuable genetic diversity in wild relatives is not found in Africa. In the case of sorghum and cowpea, intended to be grown in (or close to) its center of diversity in Africa, it was also important to consider other parts of the conceptual model that would lead to this harm, that being the likelihood of steps for genetic swamping or selective sweeps to occur. The panels agreed, based on their knowledge, that in these cases these steps in the pathway leading to a loss of genetic diversity as a resource for all three crops are also not likely. In other words, the probability of genetic swamping or selective sweeps is ‘no more likely’ than with non-GM sorghum, cowpea, or cassava counterparts.

## Considering Options for Further Testing

After reviewing the estimates of the likelihood of harm based on existing knowledge, in each case, the panels were asked to consider data gaps and ways in which they could be filled. It should be noted that in each case, the panels determined that the risk hypothesis in the first step in the pathway – ‘gene flow to wild relatives will not occur’ – could be falsified based on existing knowledge. In other words, for each crop there is some evidence to suggest that gene flow can occur between the crop and the wild relative in question. Although the existing quantification of frequency or distance of gene flow was not necessarily precise, the panels thought in each case that it would be low, and additional studies to further test the risk hypothesis, e.g., more precisely measure gene flow, would not usefully reduce uncertainty for the purposes of risk assessment.

Despite ultimately finding that the potential for harm was unlikely via any pathway, based on information considered relevant in all of the steps, each panel suggested options for experimentation to further test hypotheses derived from the pathways. These options are summarized in **Table 1**. These were hypotheses that the panels did not necessarily think ‘should be’ tested, but that ‘could be’ tested with a carefully designed experiment, although the panels stopped short of describing details of experimental designs. The relevant project team was left to decide whether to undertake these studies depending on its own priorities, including the *a priori* interpretation of what might be required in the country where the project would apply for an approval.

**Table 1:** Probability for harm related to gene flow from GM sorghum, cowpea, and cassava into wild relatives in Africa based on existing information, and options for further testing of risk hypotheses.

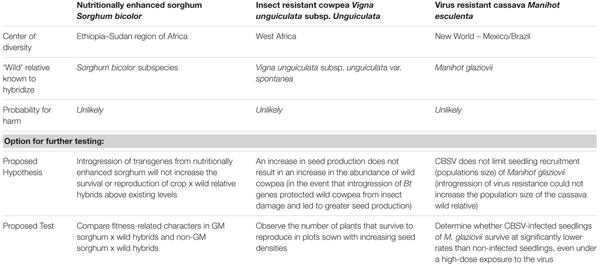

## Using Scientific Expert Panels in Risk Assessment

Expert panels proved an effective means to allow experts in different disciplines, and sometimes at odds about their initial concepts of risk, to work collegiately to organize existing knowledge into effective risk assessments. Experts in risk assessment could show how to use problem formulation and keep discussions focused on topics essential for risk assessment, while experts on gene flow, crop biology and the ecology of the wild relatives could provide the knowledge necessary to test the hypotheses arising from problem formulation. There was great opportunity for knowledge exchange: local experts could learn risk assessment methods, while risk assessment experts could learn how to integrate local ecological and agronomic knowledge into their conceptual models. Finally, expert panels excel in the ability to suggest options for further work, although this can create problems (see below).

A significant disadvantage in these expert panel deliberations, particularly such as these convened to advise the projects, was the limited input of policy to direct the scientific discussions. First, there is the problem of defining harmful effects. For the purposes of these panel discussions, the scientists on the panels had to define harm based on precedent and inference. This means that the panels may not have considered effects that some future regulator may think are important or which may be defined within specific regulatory guidelines or statutes. Conversely, in setting out what could be considered harmful effects, the opinions of the panels could inadvertently influence regulatory policy in countries where the crops are intended to be grown. Scientific expert panels are also commonly convened by the regulatory authorities. When that is the case, the regulatory policies governing the deliberations should be clear, although this remains a challenge for fledgling regulatory systems such as those in the countries targeted by the three projects discussed here. Even where there are well developed regulatory systems, harms derived from protection goals are not always well defined (Garcia-Alonso and Raybould, 2014).

The more significant challenge encountered by the absence of policy in these types of panel discussions, however, is to know whether or when a hypothesis has been tested with sufficient rigor for decision-making; that is, whether scientific uncertainty is unacceptably high and needs to be reduced. In the absence of such policy guidance, an expert scientific panel can always suggest further studies because no hypothesis can ever be proved and some uncertainty always remains. This problem is perpetuated among research scientists based on a misconception that ‘science-based’ risk assessment means ‘research-based’ risk assessment; that is, existing knowledge is never sufficient for decision-making and new studies must always be required. Having seen suggestions for further work, regulators may be reluctant to say that the work is not necessary.

Regulators to whom the remit for decision-making does fall, i.e., those responsible to use the scientific knowledge gathered for the risk assessment in order to make a decision about the acceptable level of risk, should be aware that scientific experts can inadvertently drive regulatory policy toward acquisition of new or ‘nice-to-know’ data through an emphasis on scientific uncertainty. The aim should be to maximize the use of existing knowledge and only require new data that are necessary or ‘need-to-know’ in order to reduce uncertainty to a level necessary for decision-making (see also Romeis et al., 2009). The types of complex ecological and evolutionary studies that might be designed to reduce uncertainty about the likelihood of harmful effects from gene flow, such as loss of genetic diversity in centers of diversity, are difficult to execute and are apt to lack the precision that would improve decision-making, even when precise quantitative decision-making criteria are defined by policy. An inclination to exercise excessive precaution risks a waste of scarce resources, and might even stop progress on potentially beneficial projects if the studies proposed are too costly or complex and unworkable. Yet, decision-makers will always face the challenge of balancing precaution with uncertainty. Problem formulation and hypothesis testing are useful tools to find and describe this balance.

## Conclusion

Our experience working with expert panels to inform GM crop risk assessment in centers of diversity leads us to conclude that panels such as these are valuable for gathering and organizing existing information so that it can be considered in risk assessments of GM crops, and problem formulation is a highly effective tool to facilitate this. However, these panel discussions also demonstrated that, while scientific expertise is essential in order to provide the knowledge necessary for making good decisions, science cannot operate in a policy vacuum. Risk assessors need definitions of harm, otherwise they will be forced into an almost limitless task of trying to characterize every conceivable effect of growing a particular GM crop. Furthermore, without knowledge of how decisions will be made, and in particular how the sufficiency of data will be determined, scientists will always be able to suggest new studies, and less experienced regulators may feel pressure to accept that the studies are necessary. Hence, without policy, science may produce data that are unnecessary for risk assessment and data that are not very interesting for basic research (e.g., Raybould, 2010). Suitable integration of scientific and non-scientific factors will be vital for maintaining functioning regulatory systems, especially in developing countries. With resources of all sorts often being severely limited in developing countries, clear policy is needed to ensure that they are used effectively.

## Author Contributions

KH, NE, and AR participated in the discussions described here in and wrote the manuscript.

## Conflict of Interest Statement

The authors declare that the research was conducted in the absence of any commercial or financial relationships that could be construed as a potential conflict of interest.
